# Nicotine Inhibits Cisplatin-Induced Apoptosis via Regulating α5-nAChR/AKT Signaling in Human Gastric Cancer Cells

**DOI:** 10.1371/journal.pone.0149120

**Published:** 2016-02-24

**Authors:** Yanfei Jia, Haiji Sun, Hongqiao Wu, Huilin Zhang, Xiuping Zhang, Dongjie Xiao, Xiaoli Ma, Yunshan Wang

**Affiliations:** 1 Central Laboratory, Jinan Central Hospital Affiliated to Shandong University, Jinan, China; 2 College of Life Science, Shandong Normal University, Jinan, China; Duke University Medical Center, UNITED STATES

## Abstract

Gastric cancer incidence demonstrates a strong etiologic association with smoking. Nicotine, the major component in tobacco, is a survival agonist that inhibits apoptosis induced by certain chemotherapeutic agents, but the precise mechanisms involved remain largely unknown. Recently studies have indicated that α5-nicotinic acetylcholine receptor (α5-nAChR) is highly associated with lung cancer risk and nicotine dependence. Nevertheless, no information has been available about whether nicotine also affects proliferation of human gastric cancer cells through regulation of α5-nAChR. To evaluate the hypothesis that α5-nAChR may play a role in gastric cancer, we investigated its expression in gastric cancer tissues and cell lines. The expression of α5-nAChR increased in gastric cancer tissue compared with para-carcinoma tissues. In view of the results, we proceeded to investigate whether nicotine inhibits cisplatin-induced apoptosis via regulating α5-nAChR in gastric cancer cell. The results showed that nicotine significantly promoted cell proliferation in a dose and time-dependent manner through α5-nAChR activation in human gastric cells. Furthermore, nicotine inhibited apoptosis induced by cisplatin. Silence of α5-nAChR ablated the protective effects of nicotine. However, when co-administrating LY294002, an inhibitor of PI3K/AKT pathway, an increased apoptosis was observed. This effect correlated with the induction of Bcl-2, Bax, Survivin and Caspase-3 by nicotine in gastric cell lines. These results suggest that exposure to nicotine might negatively impact the apoptotic potential of chemotherapeutic drugs and that α5-nAChR/AKT signaling plays a key role in the anti-apoptotic activity of nicotine induced by cisplatin.

## Introduction

Gastric cancer is one of the major causes of cancer deaths in the world. Apparently, both genetic and environmental factors are involved in gastric carcinogenesis. Previous findings have unraveled the strong association between cigarette smoke and gastric cancer incidence [1–3], however, the detailed mechanism has not been fully studied.

Nicotine, a major component of cigarette smoke, has been shown to be involved in the initiation, promotion, and even progression of several tumors including gastric cancer. Several lines of evidence suggest that nicotine exerts its cellular functions through nicotinic acetylcholine receptors (nAChRs). Different epithelial cells, not only neuron cells, express nAChRs and the structure of nAChRs is a homo-(α7or α9) or heteropentamer (α2–α10; b2–b4). Studies have demonstrated that nicotine stimulated the proliferation of human gastric cancer cells through its interaction with α7-nAChR [4, 5]. While different members of nAChR family may regulate converging signaling pathways, they often have diverse and even opposing actions. Recently, genome wide association studies have indicated that α5-nAChR is highly associated with lung cancer risk and nicotine dependence [6, 7]. Nevertheless, no information has been available about whether nicotine also affects proliferation of human gastric cancer cells through regulation of α5-nAChR.

The delivery of chemotherapeutic agent cisplatin following surgical resection currently defines the standard treatment for gastric cancer [8–10]. Unfortunately, acquired resistance to cisplatin is common and evasion of cell apoptosis is recognized as one of the major mechanisms responsible for cisplatin resistance [11, 12]. In particular, nicotine could inhibit apoptosis induced by cisplatin in lung cancer cells [13–15] and oral cancers [16]. The inhibitory effect of nicotine on apoptosis has been attributed to its ability to activate anti-apoptotic proteins like Bcl-2 and Survivin, as well as inactivation of proapoptotic proteins like Bax and Caspase-3, through the activation of both PI3K/AKT and PKC/ERK signaling pathways in cancer cells [13–15]. This effect of nicotine on cell apoptosis is also mediated by nAChRs, but in addition to α5-nAChR, other subunits seem to be involved [17, 18].

Although many of these mechanisms have been observed in lung cancer [19–21], there is no evidence of the anti-apoptotic effect and the mechanism exerted by nicotine on gastric cancer cells. The aim of the present study was to investigate nicotine inhibits cisplatin-induced apoptosis via regulating α5-nAChR in gastric cancer cell. Moreover, we propose the involvement of pro-survival factors, such as Bcl-2 and Survivin, activated by AKT pathways, respectively.

## Materials and Methods

### Ethics Statement

The study protocol was approved by the Medical Ethics and Human Clinical Trial Committee of the Jinan Central Hospital. Written informed consent was obtained from all patients.

### Tissue specimens, Cell culture and drug treatment

Fifty formalin-fixed, paraffin-embedded samples containing 40 specimens of gastric cancer and 10 para-carcinoma tissues were retrospectively and randomly selected from the files of the Jinan Central Hospital after the protocol was approved by the local research ethics committee. According to the record of smoking (or not) in the case history of the patients, 32 cases had no smoking intake history, 8 had smoking intake history. All the samples were evaluated for diagnosis by two experienced pathologists for diagnosis.

The human gastric cancer cell lines MKN28, SGC7901, BGC823, MGC803, AGS, HGC27, and MKN45 were obtained from the Cell Bank of Shanghai, Institute of Biochemistry and Cell Biology, Chinese Academy of Sciences. Cells were cultured in DMEM (Invitrogen) supplemented with 10% fetal bovine serum (FBS; Invitrogen), 1% antibiotics at 37°C in 5% CO2 humidified air. In all experiments, 60–70% of confluent cells were washed and incubated in serum-free medium for 24 hours prior to treatment with nicotine, cisplatin and LY294002 (Sigma, St. Louis, MO) for the indicated time.

### Immunohistochemistry

Immunohistochemical staining using the streptavidin peroxidase method (S-P method) was performed on 4 μm sections of paraffin-embedded specimens to detect α5-nAChR expression in gastric cancer and para-carcinoma tissues. In brief, after deparaffinization and hydration, the slides were treated with endogenous peroxidase in 0.3% H_2_O_2_ for 30 min and blocked for 2 h at room temperature with 1.5% blocking serum in phosphate-buffered saline (PBS). The sections were then incubated with anti-α5-nAChR antibody (Abcam, Inc. Cambridge, MA) (1:100 dilution) at 4°C overnight, washed with PBS, and incubated with secondary anti-mouse biotinylated antibody (KIT-5010, Max Vision, Maixin.Bio, China) (1:2000) in PBS for 30 min at 37°C. The antibody binding was detected using the streptavidin–biotin–peroxidase complex/HRP (Code K0377; Dako) with 3, 3- diaminobenzidine for 3 min as a chromogenic substrate. The slides were then lightly counterstained with hematoxylin. As a negative control, duplicate sections were stained without exposure to the primary antibodies. The results were observed under a microscope.

### Quantitative Real-time RT-PCR

Total RNA was isolated using the Trizol reagent (Invitrogen) according to the manufacturer’s instructions. Twenty-five nanogram total RNA per sample was reverse transcribed by using the Reverse Transcription Reaction Kit (Takara Code: DRR061S) according to the manufacturer's instructions. Quantitative real-time PCR was performed analyzed on the Applied Biosystems 7300 Real-Time PCR System to determine the relative amounts of α5-nAChR and β-actin (internal control) mRNAs expressed. The SYBR Green Supermix was used for all real-time PCR reactions. PCR primers used in this study are as follows: α5-nAChR Forward: GAAACTGAGAGTGGTAGTGGACCAA, Reverse: GGGCTATGAATTTCC AATCTTCAAC; glyceraldehyde 3-phosphate dehydrogenase (GAPDH) forward, 5’-CATGAGAAGTATGACAACAGCCT-3’ and reverse, 5’-AGTCCTTCCACG ATACCAAAGT-3’. The quantitative real-time PCR parameters were 95°C for 10s as a pre-denature step followed by 40 PCR cycles of 95°C for 5 s, 60°C for 30 s and 72°C for 10 min. All the samples were performed in triplicates in each experiment. The relative amount mRNA was calculated using the comparative CT method after normalization to β-actin mRNA levels.

### Western blotting analysis

Cell pellets were homogenized in extraction buffer (50 mmol/L Tris-HCl, pH 6.8, 0.1% SDS, 150 μmol/L NaCl, 100 mg/L phenylmethylsulfonyl fluoride, 1 mg/L aprotinin, 1% NP-40 and 0.5% sodium orthovanadate), incubated at 4°C for 30 min, and centrifuged for 20 min at 12000 g/min. Total protein in the cell lysate was measured with use of the Bio-Rad colorimetric kit (Bio-Rad, Hercules, CA, USA). For western blot analysis, total protein was separated on 10% SDS-PAGE and transferred onto nitrocellulose membranes (0.45 μm, Millipore, Billerica, MA, USA), which were incubated for 24 h at 4°C with the antibodies for α5-nAChR (1:500, ab41173 or ab166718), AKT(1:500, Epitomics Cat no:1085–1), P-AKT(1:500, Epitomics Cat no:2118–1), Caspase-3 (1:500, Epitomics Cat no:1087–1), Bcl-2 (1:500, Epitomics Cat no:1017–1), Survivin (1:500, Epitomics Cat no:2463–1) and GAPDH (1:1000; ab37168), then horseradish peroxidase-conjugated anti-mouse/rabbit IgG antibody (Santa Cruz Biotechnology) after a final wash. Signals were detected with use of an enhanced chemiluminescence kit (Amersham Pharmacia, Buckinghamshire, UK). GAPDH level was an internal standard.

### RNA interference

A double strand siRNA oligonucleotide targeting CHRNA5, which encodes α5-nAChR, (sense: 5’-CCCGCAAACUACAAAAGUUTT-3’, antisense: 5’-AACUUUUCUAGUUUGCCGGTG-3’) was synthesized by Shanghai Genepharma Co. Ltd. (China). A pair of negative control siRNA was also designed with sequences different from siRNA-CHRNA5 and not homologous to any sequences found in gene bank (sense: 5’-UUCUCCGAACGUGUCACGUTT-3’, antisense: 5’-ACGUGACACGUUCGGAGA-3’). The cells were plated in six-well plates. When cells reached 30–50% confluence, the siRNAs were added to a final concentration of 50 nM with lipofectamine 2000 (Invitrogen) according to the manufacturer's instruction.

### Cell Viability Assay

Cell viability was determined by the CCK8 assay (Dojindo, Tokyo, Japan). Briefly, cells plated in 96-well plates (1500 cells/well) were treated with nicotine at the indicated doses. The cell proliferation assay was performed by the addition of 10 μl CCK8 solution to each well, followed by incubation at 37°C for 2 h. Absorbance was measured at a wavelength of 450 nm using a microplate reader (Synergy 2 Multi-Mode Microplate Reader; BioTek, Winooski, VT, USA).

### Annexin V/7-AAD staining

BGC823 cells were cultured at confluence into 6-well tissue culture plates (Falcon, Becton Dickinson Labware) in a complete medium. Then the medium was replaced by fresh complete medium or by serum free medium; the cells were then stimulated with 100 μM nicotine alone or in combination with 20 mM cisplatin for 24 h based on our previous data, trypsinized and washed twice with PBS. The cells were stained with PE labeled annexin V/7-AAD (7-aminoactinomycine-D) according to the instructions of the manufacturer (annexin V/7-AAD kit; Becton, Dickinson and Company). Briefly, a washed cell pellet was resuspended in 500 μl binding buffer. Next, 5 μl Annexin-V-PE and 5μl 7-AAD were added. Flow cytometric analysis was performed immediately after supravital staining. Data acquisition and analysis were performed in a Becton Dickinson FACSCalibur flow cytometer using CellQuest software. The cells in early stages of apoptosis were AnnexinV positive and 7-AAD negative, whereas the cells in the late stages of apoptosis were both AnnexinV and 7-AAD positive.

### Hoechst 33342 staining

Cells were seeded in 12-well tissue culture plates and treated with the indicated concentrations of nicotine and cisplatin. At the end of each incubation, cells were fixed with 4% paraformaldehyde for 20 min, washed with PBS, and then incubated with Hoechst 33342 (1 μg/ml) for 10 min. After washing with PBS, cells were observed using a fluorescent microscope (Olympus, Japan). At least 400 cells from 12 randomly selected fields per dish were counted, and each treatment was performed in triplicate.

### Statistics analysis

The result of the immunohistochemistry was analyzed using the χ^2^ test. All results were presented as means ± S.D. from triplicate experiments performed in a parallel manner unless otherwise indicated. The significance of difference between control groups and nicotine treatment groups was determined by a two-tailed Student’s t-test. Differences were considered significant at P < 0.05 or P < 0.01.

## Results

### α5-nAChR expressions in gastric cancer tissue specimens and cell lines

**Fig 1:**
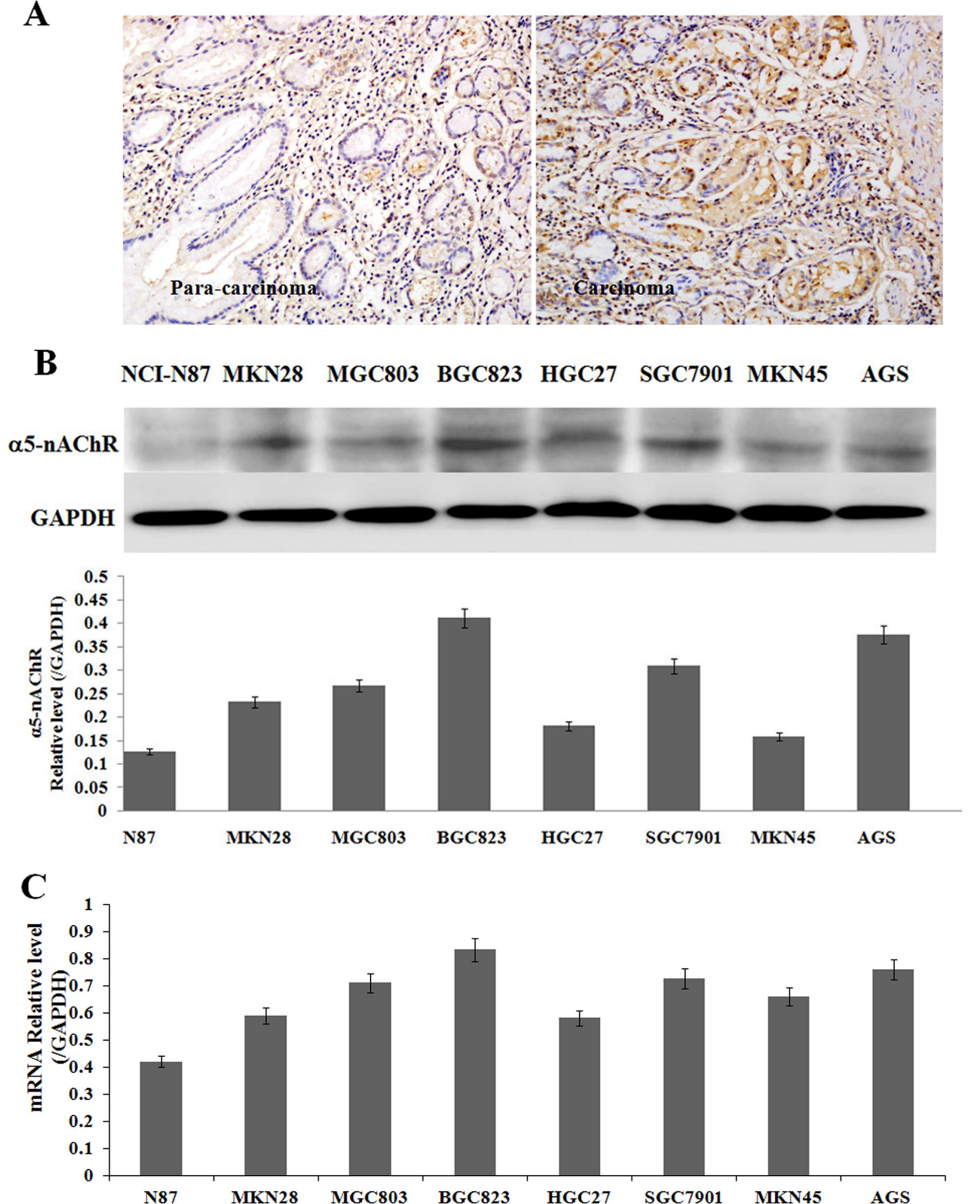
The expression of α5-nAChR in gastric cancer tissues and cell lines. A: Expression of α5-nAChR in gastric para-carcinoma tissue (Left) and cancer Tissues (Right). (IHC 200×); B: Protein expression of α5-nAChR in gastric cancer cell lines (N87, MKN28, MGC803, BGC823, HGC27, SGC7901, MKN45, AGS); C: The expression of α5-nAChR mRNA in the above cells in (A) was analyzed by RT-PCR, and normalized to that of GAPDH.

The expression of α5-nAChR were detected and localized in paraffin-embedded human gastric tissue sections. α5-nAChR was mainly localized on the membrane of the tumor cells, although some cytoplasm staining was also observed (Fig 1A). Expression of α5-nAChR was higher in gastric cancer tissues (77.5%, 31/40) than that in para-carcinoma tissues (20%, 2/10). The results showed a trend that the positive rate of α5-nAchR expression increased in patients with nicotine intake history (87.5%, 7/8) compared with patients without nicotine intake history (75.0%, 24/32).

α5-nAChR mRNA and protein were detectable in a panel of gastric cancer cell lines. The cell lines expressed different levels of α5-nAChR protein, where cell lines with a higher expression were BGC823, AGS and SGC7901 normalized to that of GAPDH(Fig 1B). The levels of α5-nAChR mRNA expression detected by RT-PCR correlated with the levels of protein expression (Fig 1C). BGC823 expresses higher levels of α5-nAChR than that of other cell lines. For further functional experiments, BGC823 cell line was chosen due to its higher expression (suitable for induction by nicotine or silencing by siRNA).

### Nicotine promoted gastric cancer cell proliferation through α5-nAChR

**Fig 2:**
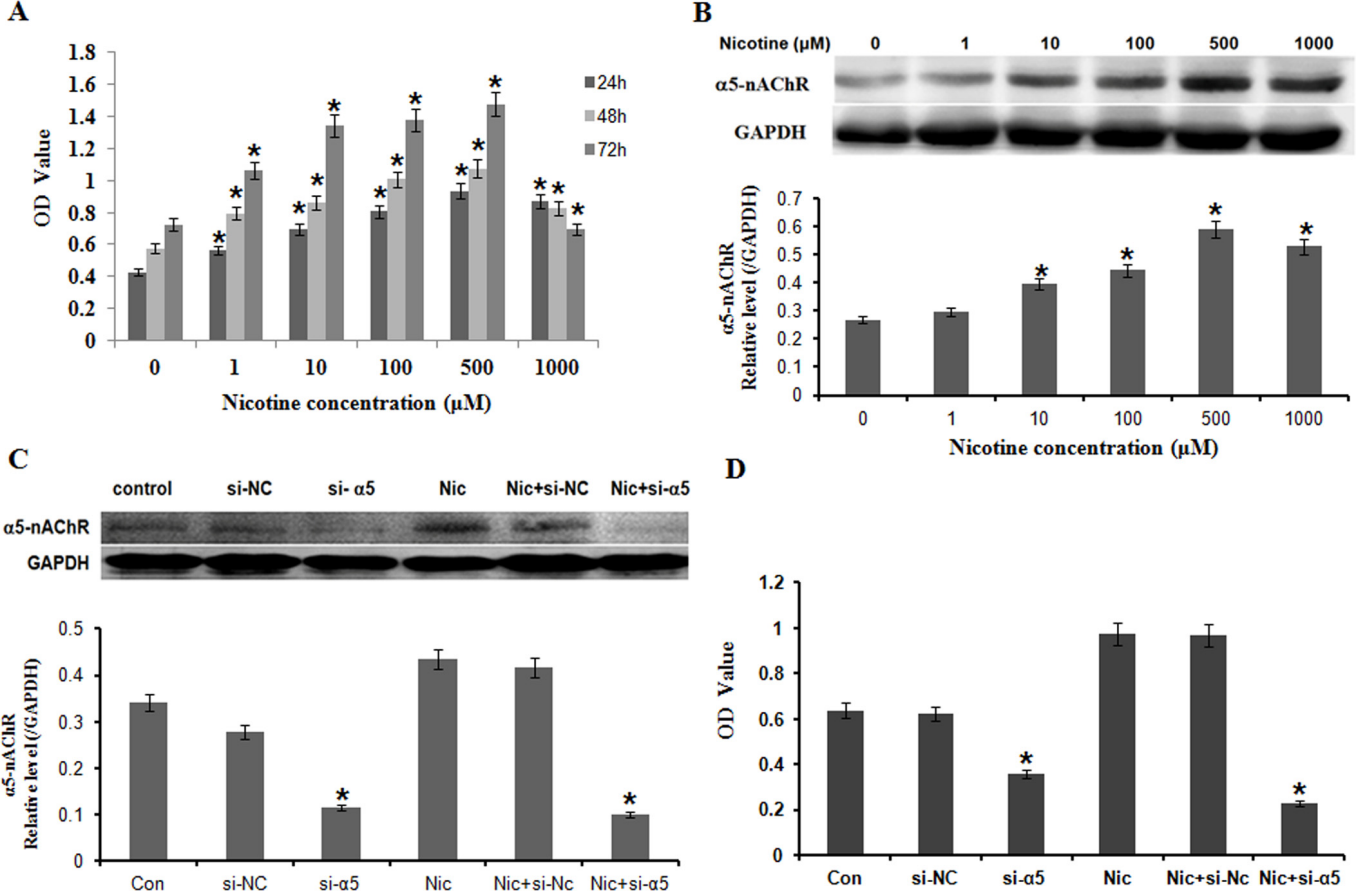
Nicotine promoted BGC823 cell proliferation through α5-nAChR. A: BGC823 were exposed to nicotine (0, 1, 10, 100, 500, 1000μM) for 24, 48, and 72h, respectively. Nicotine promoted cell proliferation in a time-dependent and concentration-dependent relation; B: Treatment of nicotine at the dose of nicotine (0, 1, 10, 100, 500, 1000μM) for 24 hours promoted the expression of α5-nAChR protein in a dose-dependent manner; C: Cells were transfected with α5-nAChR specific siRNA (si-α5) and then treated with nicotine at 100μM for 24 hours. α5-nAChR expressions were significantly decreased compared with scrambled nonspecific control siRNA (si-NC) and Nic (Nicotine) + si-NC group; D: By comparison with the si-NC, transfection with si-α5 considerably inhibited cell proliferation. *P < 0.05 vs. the untreated control; each experiment was performed in triplicate.

To determine whether nicotine may regulate the proliferation of gastric cells, we examined the effect of nicotine with various concentrations on cell viability in BGC823 by a CCK8 analysis (Fig 2A). BGC823 were exposed to nicotine (0, 1, 10, 100, 500, 1000 μM) for 24, 48, and 72h, respectively. As shown in Fig 2A, nicotine promoted cell proliferation in a time-dependent and concentration-dependent relation. Nicotine significantly stimulated cell proliferation at lower concentration (1, 10, 100, 500 μM) and time (24–72 h), but a noticeable decrease in cell number was observed in nicotine-treated cultures at higher concentration (1000 μM) and time (72 h). Studies showed levels of nicotine in the body vary widely among individuals even when smoking the same number of identical cigarettes [22–24].The concentration of nicotine (100 μM) used in the present study mimicked the daily intake of cigarettes in moderate smokers [25, 26]. The concentration of nicotine (100 μM) is equivalent to concentration in the saliva in smoker who intake 25 cigarettes/day [27]. For further functional experiments, the concentration of nicotine (100 μM) was chosen to treat gastric cells for 24h.

To determine whether the nicotine-mediated promotion of cell proliferation in gastric cells is mediated through α5-nAChR signaling, we analyzed the effect of nicotine on the expression of α5-nAChR protein in BGC823 cells by Western blot. As shown in Fig 2B, treatment of nicotine at the dose of nicotine (0, 1, 10, 100, 500, 1000 μM) for 24 hours promoted the expression of α5-nAChR protein in a dose-dependent manner. To further confirm the involvement of α5-nAChR signaling pathway in the nicotine-mediated promotion of gastric cancer cell proliferation, we blocked α5-nAChR protein expression by transfecting BC823 cells with a α5-nAChR-specific siRNA (si-α5-nAChR) (Fig 2C) and evaluated its effects on nicotine-mediated promotion of cell proliferation by CCK assay (Fig 2D). By comparison with the scrambled nonspecific control siRNA (si-NC), transfection with si-α5-nAChR inhibited cell proliferation. Moreover, si-α5-nAChR transfection significantly reduced the nicotine-mediated promotion of cell proliferation in BGC823 cells (Fig 2D).

### Nicotine inhibition of cisplatin-induced apoptosis

**Fig 3:**
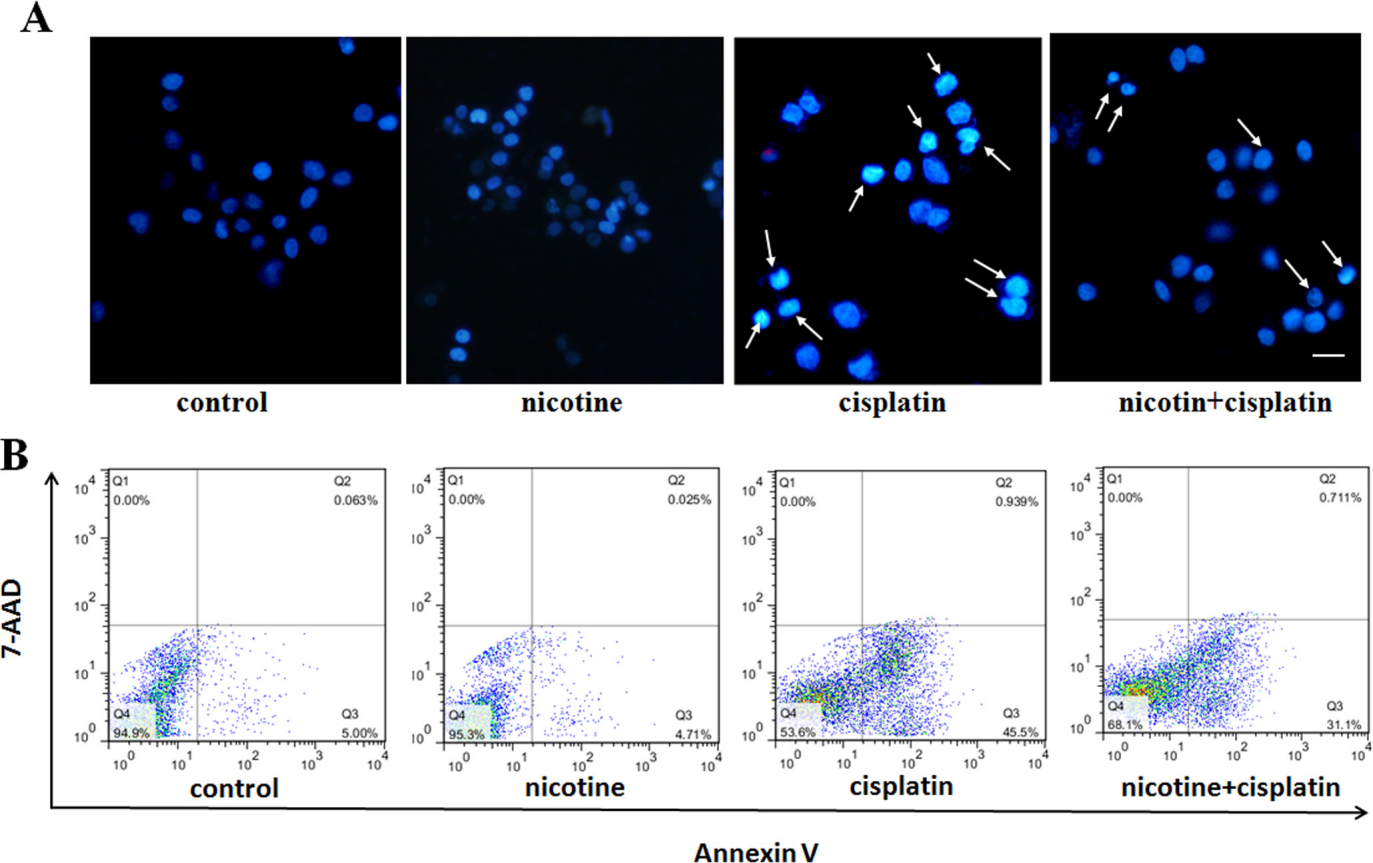
Nicotine inhibition of cisplatin-induced apoptosis. A: Cisplatin induced cells apoptosis compared with the control. In contrast, the cells co-treated with 100 μM nicotine and 20 mM cisplatin showed little nuclear fragmentation and few apoptotic bodies by fluorescence microscopy (200 X); B: Cisplatin induced apoptosis was assayed by AnnexinV/7-AAD staining. Flow cytometry plots showed that the proportion of total apoptotic cells was decreased when exposed to 20 mM cisplatin combined with 100 μM nicotine.

To further characterize the apoptosis effects of nicotine combined with cisplatin on BGC823, cisplatin was used to induce apoptosis in the BGC823 cells. The cells were treated with 20 mM cisplatin [28]. As shown in Fig 3A, the apoptotic cells became rounded in shape and their nuclei exhibited a fragmented morphology, forming apoptotic bodies. In contrast, the cells co-treated with nicotine and cisplatin showed little nuclear fragmentation and few apoptotic bodies.

Cisplatin induced apoptosis was further assayed by AnnexinV/7-AAD staining. Flow cytometry plots showed that the proportions of total apoptotic cells (AnnexinV+/7-AAD−) decreased from43.2±2.32% to29.9 ± 1.26%, when exposed to cisplatin combined with 100 μM nicotine (Fig 3B). These results suggested that nicotine can suppress apoptosis induced by cisplatin.

### α5-nAChR/AKT signaling pathway involved in anti- apoptotic effects of nicotine in cisplatin-induced apoptosis of BGC823 cells

**Fig 4:**
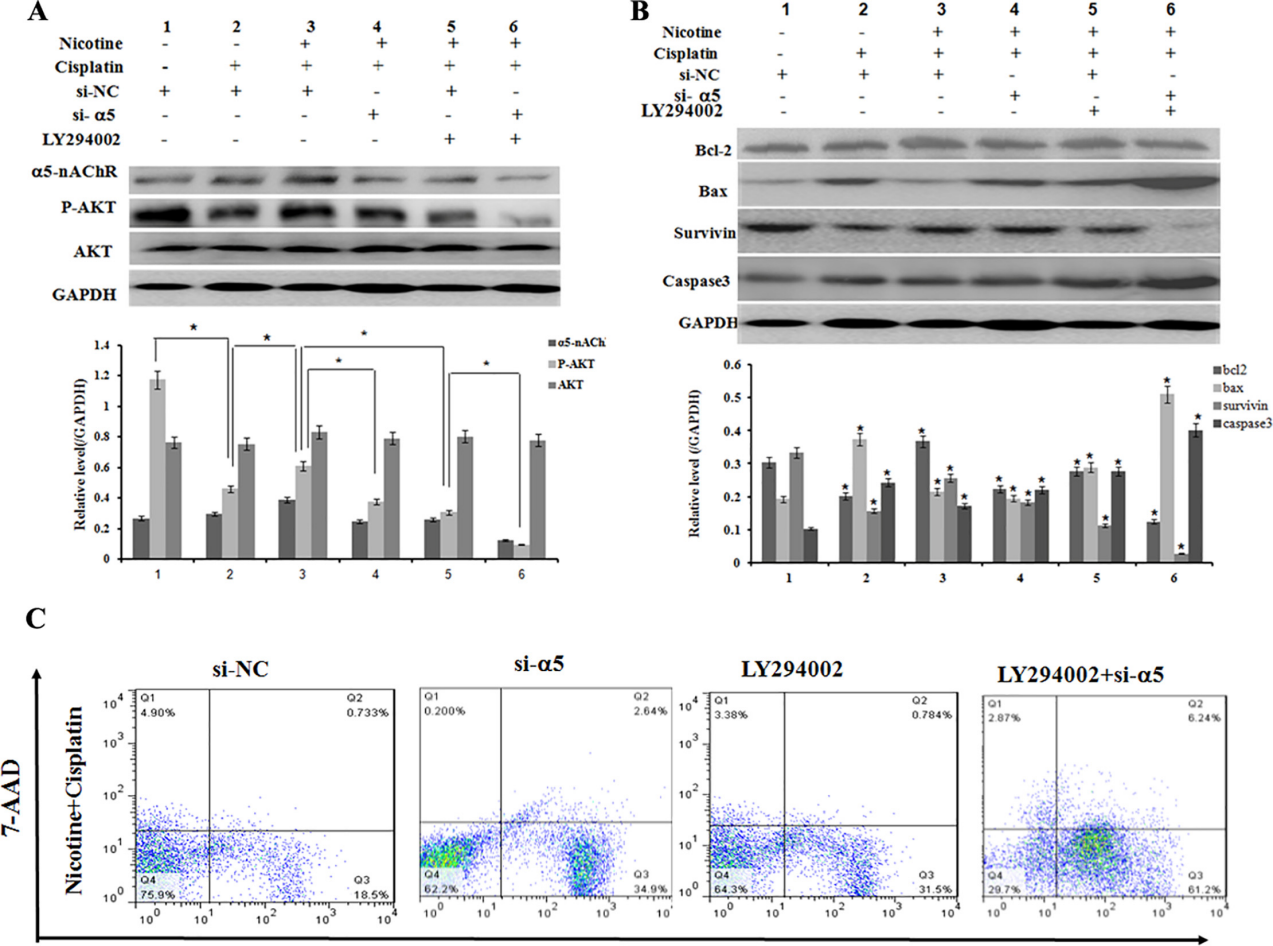
α5-nAChR/AKT signaling involved in anti-apoptotic effects of nicotine in cisplatin-induced apoptosis of BGC823 cells. A: P-AKT was activated after exposure to 100μM nicotine in BGC823 cells (lane 2 and lane3). Cisplatin strongly suppressed activity of P-AKT (lane 1 and lane 2) but nicotine also induced P-AKT in the presence of cisplatin (lane 2 and lane 3). Down-regulation of α5-nAChR expression decreased the level of P-AKT (lane 4 and lane 5). Treatment with LY294002 downregulated P-AKT expression (lane 3 and lane 5). Combination LY294002 with si-CHRNA5 transfection significantly repressed the nicotine induced P-AKT protein levels (lane 3 and lane 6); *p<0.05; B: Cisplatin induced an increase in caspase-3 and Bax activation in BGC823 cells a decrease in Bcl-2 and Survivin expressions, whereas nicotine blocked cisplatin- induced Bcl-2, Bax, caspase-3 and Survivin expressions; With silence of α5-nAChR co-administrated LY294002, an increased apoptosis was observed with the induction of Bcl-2, Bax, Survivin and Caspase-3 by nicotine in BGC823 cells. *p<0.05; C: Assessment of apoptosis by AnnexinV/7-AAD staining in each group. BGC823 cells were pre-treated with 100 μM nicotine and cisplatin for 24 h, and/or si-α5-nAChR for 48h, and/or AKT inhibitor LY294002 for 24h, and harvested.

To determine the role of AKT in mediating the effects of nicotine in these cells, we determined whether nicotine induces activation of AKT in BGC823 cells. In Fig 4A, 20 mM cisplatin strongly suppressed activity of AKT (lane 2) but nicotine increased AKT expression (lane 3) in the presence of cisplatin. It suggested that AKT was activated after exposure to 100 μM nicotine in BGC823 cells as reported in various papers [29, 30]. The down-regulation of α5-nAChR expression decreased P-AKT expression (lane 4). Treatment with 10 mM LY294002, a phosphatidylinositol 3-kinase (PI3K)/AKT pathway inhibitor, decreased the anti-apoptotic effects of nicotine in BGC823 cells (lane 5). In addition, treatment with LY294002 combined with si-CHRNA5 transfection significantly repressed the nicotine induced P-AKT protein levels (lane 6). These data suggest that α5-nAChR /AKT pathways play a critical role in mediating the effects of nicotine and chemotherapy in gastric cancer cells.

We further examined the effects of nicotine on cisplatin-induced caspase-3 activation in BGC823 cells by Western blot assay. As shown in Fig 4B, cisplatin induced an increase in caspase-3 activation, whereas nicotine blocked cisplatin-induced caspase-3 activation in BGC823 cells. Moreover, exposure of BGC823 cells to cisplatin caused the expression of apoptotic protein Bax increased while the prosurvival protein Bcl-2 and Survivin decreased, which were inhibited by treatment with nicotine.

Meantime, as shown in Fig 4C, the anti-apoptotic effect of nicotine was obviously blocked by si-α5-nAChR in BGC823 cells. Flow cytometry plots showed that the proportion of total apoptotic cells (AnnexinV+/7-AAD−) increased from 18.5 ± 0.92% to 34.9 ± 1.74% after si-5 treatment compared with si-NC group. Addition of AKT inhibitor LY294002 with si-α5-nAChR significantly promoted the apoptotic effect of cisplatin from 31.5 ± 1.57% to 61.2 ± 3.06% compared with LY294002 group. These results indicate that nicotine plays an important role in the prevention of cisplatin-induced apoptosis via α5-nAChR/AKT signaling pathway.

## Discussion

This study is the first to demonstrate a vital role of α5-nAChR in nicotine anti-apoptosis of cisplatin in human gastric cancer cells. The analysis of clinical specimens indicated that α5-nAChR expression is generally higher in tumor cells compared with para-carcinoma cells. The results showed a trend that the positive rate of α5-nAchR expression was higher in patients with nicotine intake history than patients without nicotine intake history. The in vitro results showed that nicotine up-regulated the expression of α5-nAChR protein and inhibited cisplatin-induced apoptosis by regulating α5-nAChR/AKT signaling pathway in gastric cancer cells. Nicotine prevented cisplatin-induced activation of caspase-3 and Bax, and up-regulated the expression of anti-apoptotic proteins, Bcl-2 and Survivin. Furthermore, activation of AKT was found to play a role in mediating cisplatin-induced apoptosis, as well as anti-apoptotic effects of nicotine in BGC823 cells. It may be interesting to pay attention to the relationship between cigarette smoke (second smoking) and gastric cancer incidence.

Previous findings have unraveled the strong association between cigarette smoke and gastric cancer [31, 32]. Nicotine was shown to increase the proliferation and migration of gastric cancer cells by inducing cyclooxgenase-2 (COX-2), prostaglandin E2, VEGF, β-adrenoceptors, protein kinase C [26, 33, 34]. These effects appear to be mediated through the homopentameric α7-nAChRs [35]. However, recent studies have demonstrated an unexpected effect of the heteropentameric α5-nAChRs on nicotine intake [36–38]. Our previous works reported that the nicotine/α5-nAChR pathway is critical for lung cancer cell viability [21, 39]. Nevertheless, no information has been available about whether nicotine also affects proliferation of human gastric cancer cells through regulation of α5-nAChR. Here, we studied the role of α5-nAChR in human gastric cancer. Our results demonstrated that expression of α5-nAChR was higher in gastric cancer tissues compared to para-carcinoma tissue, which suggested that α5-nAChR may play a role in gastric carcinogenesis. Furthermore, nicotine promoted α5-nAChR protein expression and blocking the activation of α5-nAChR by siRNA attenuated nicotine-induced gastric cancer cell proliferation. It suggested that α5-nAChR is one of the major molecules to mediate cell proliferation stimulated by nicotine in gastric cancer cells.

The current standard chemotherapy for gastric cancer is cisplatin, but the success rate of this treatment is poor. The efficacy of cisplatin depends on the ability to induce DNA damage [40, 41]. Hence, how cancer cells respond to cisplatin- induced apoptosis plays a critical role in cisplatin sensitivity. Recent reports show that nicotine inhibits cisplatin induced apoptosis in lung cancer cells, oral cancer, Raw264.7 and El4 cells [13, 16, 18], which may suggest that nicotine has the ability not only to promote cancer development by activating cell growth pathways, but also to reduce the efficacy of chemotherapeutic agents by stimulating survival pathways. In agreement with the previous findings, results from the present study showed that co-treatment of the cells with cisplatin and nicotine, a significant activation of caspase-3 was observed, indicating that nicotine is able to determine an inhibition of the apoptotic potential of the cisplatin in gastric cancer.

The signaling pathways that regulate cell processes, including cell proliferation, cell cycle progression and cell apoptosis, have significant impact on deciding cellular response to cisplatin. Previous studies have shown that activation of AKT pathway may lead to resistance to cisplatin [42, 43]. An over-expression of serine phosphorylated AKT has been detected in a wide range of human cancers, including gastric cancer [44, 45]. As reported in various papers, nicotine by binding to nAChR leads to downstream activation of tumor promoting proteins including activation of the AKT pathways [46–48]. Exposure to nicotine might negatively impact on the apoptotic potential of cisplatin in human oral cancer cells, and the AKT pathway was required for nicotine function [16]. However, AKT pathway of nicotinic downstream signaling and the anti-apoptotic effects of nicotine in gastric cancer cells were unknown. Therefore, we investigated whether P-AKT may account for the antagonizing effect of nicotine on the cytotoxicity of cisplatin. We found that co-treated with siRNA-α5-nAChR and LY294002, a PI3k/AKT pathway inhibitor, increased cisplatin-induced apoptosis and attenuated the effects of nicotine in BGC823 cells. Several studies highlighted nicotine activated AKT signaling pathways is in modulating cell proliferation and survival through activation of the prosurvival protein Bcl-2 and Survivin [15, 49, 50]. Our research also demonstrated that role of nicotine on cell apoptosis induced by cisplatin through α5-nAChR/AKT is confirmed by induction of Survivin and Bcl-2, as final effectors of the pathways above.

**Fig 5:**
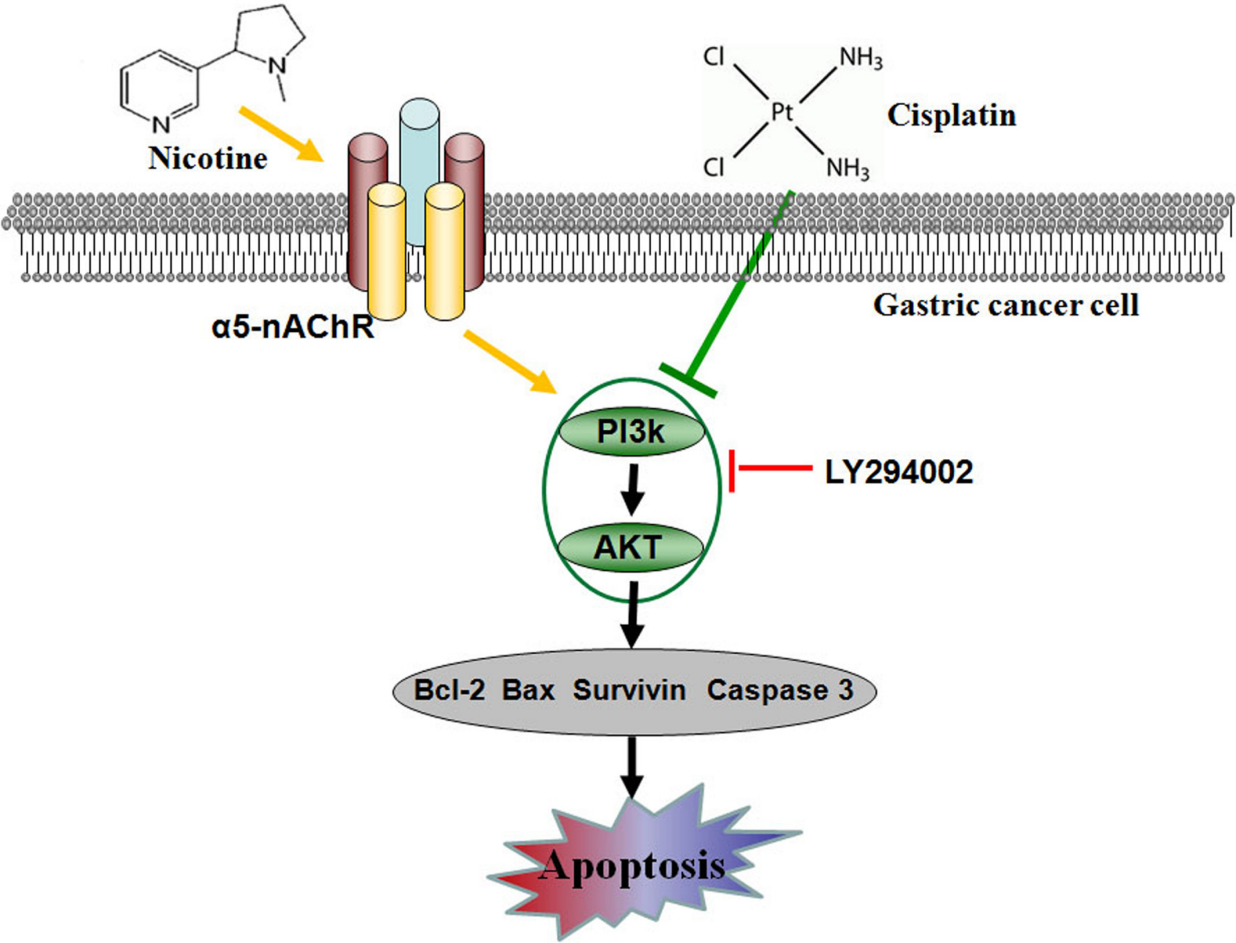
Schematic diagram of nicotine-mediated α5-nAChR/AKT signaling pathway in the prevention of cisplatin-induced apoptosis in gastric cancer. Nicotine may interact with α5-nAChR on the surface of gastric cancer cells, then activate AKT signaling pathway and up-regulate Survivin and Bcl-2 expressions to prevent cisplatin-induced apoptosis.

In summary, we demonstrated that nicotine activated α5-nAChR/AKT signaling and is involved in the resistance of cisplatin in gastric cancer. These findings provide new insights into the possible molecular mechanisms of nicotine inhibition of cisplatin-induced apoptosis in human gastric cancer cells (Fig 5). Nicotine present in cigarette smoke may interfere with gastric cancer pharmacological treatment by inhibiting chemotherapeutic drug-induced apoptosis. Strategies aimed at understanding nicotine-mediated signaling may facilitate the development of improved therapies in gastric cancer.

## Supporting Information

S1 Fig Expression of α5-nAChR and α7-nAChR without or with α5-siRNA treatment. (TIF)

S2 Fig Down-regulation of α5-nAChR expression decreased the level of P-AKT. (TIF)

S3 Fig Expression of α5-nAChR in BGC823 cells without or with si-α5-nAChR treatment. (TIF)

S4 Fig Nicotine promoted BGC823 cell proliferation through α5-nAChR and α7-nAChR. (TIF)

S5 Fig Nicotine promoted SGC7901 cell proliferation through α5-nAChR. (TIF)

S6 Fig Nicotine inhibition of cisplatin-induced apoptosis of SCG7901. (TIF)
