# Supplementary figures and images for: Nicotine Inhibits Cisplatin-Induced Apoptosis via Regulating α5-nAChR/AKT Signaling in Human Gastric Cancer Cells
Source: PLoS One. 2016 Feb 24;11(2):e0149120. doi: 10.1371/journal.pone.0149120 (PMC4765889; doi:10.1371/journal.pone.0149120)

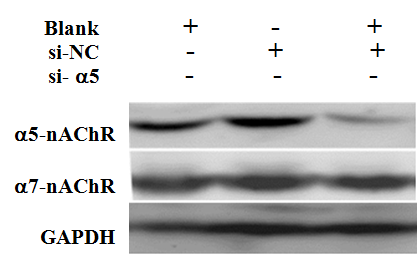

Supplement: S1 Fig — (TIF) [file pone.0149120.s001.tif]

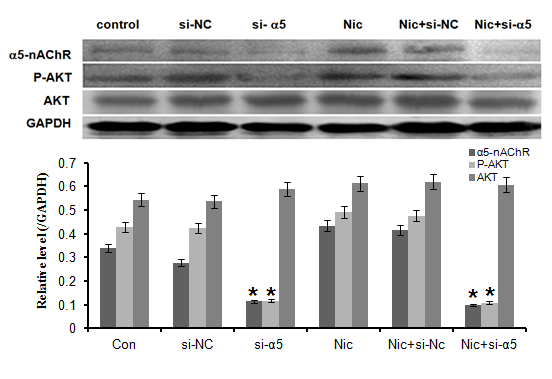

Supplement: S2 Fig — (TIF) [file pone.0149120.s002.tif]

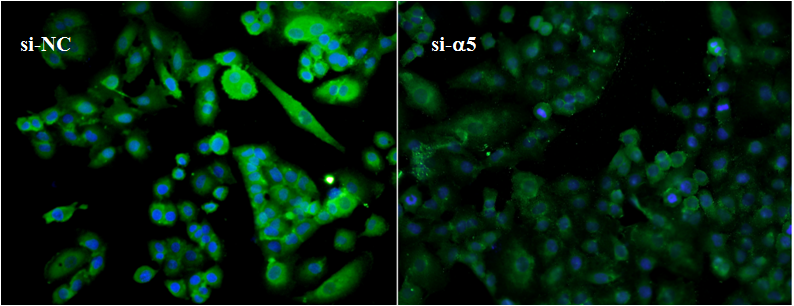

Supplement: S3 Fig — (TIF) [file pone.0149120.s003.tif]

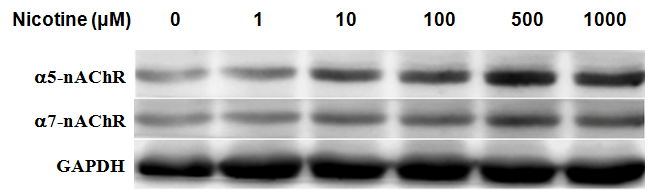

Supplement: S4 Fig — (TIF) [file pone.0149120.s004.tif]

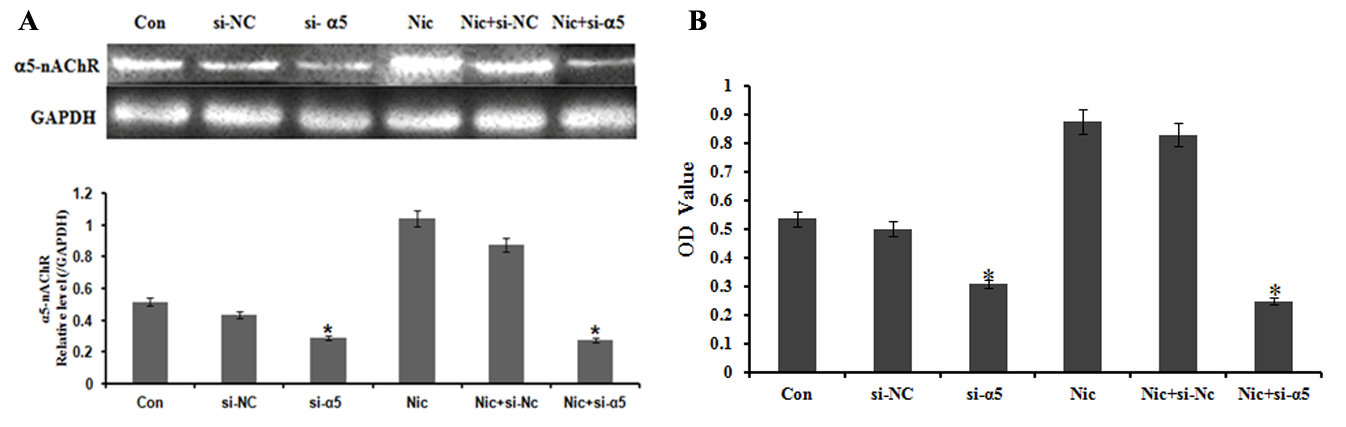

Supplement: S5 Fig — (TIF) [file pone.0149120.s005.tif]

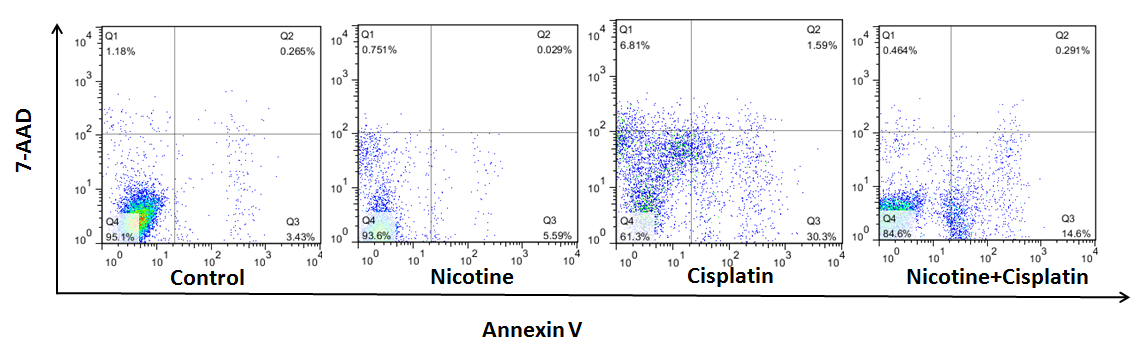

Supplement: S6 Fig — (TIF) [file pone.0149120.s006.tif]
